# *De Novo* Sequencing and Transcriptome Analysis of *Wolfiporia cocos* to Reveal Genes Related to Biosynthesis of Triterpenoids

**DOI:** 10.1371/journal.pone.0071350

**Published:** 2013-08-14

**Authors:** Shaohua Shu, Bei Chen, Mengchun Zhou, Xinmei Zhao, Haiyang Xia, Mo Wang

**Affiliations:** 1 College of Plant Science and Technology, Huazhong Agricultural University, Wuhan, Hubei, People’s Republic of China; 2 Shanghai Institute of Plant Physiology and Ecology, Chinese Academy of Science, Shanghai, People’s Republic of China; University Paris South, France

## Abstract

*Wolfiporia cocos* Ryvarden *et* Gilbertson is a saprophytic fungus in the Basidiomycetes. Its dried sclerotium is widely used as a traditional crude drug in East Asia. Especially in China, the dried sclerotium is regarded as the silver of the Chinese traditional drugs, not only for its white color, but also its medicinal value. Furthermore, triterpenoids from *W. cocos* are the main active compounds with antitumor and anti-inflammatory activity. Biosynthesis of the triterpenoids has rarely been researched. In this study, the *de novo* sequencing of the mycelia and sclerotia of *W. cocos* were carried out by Illumina HiSeq 2000. A total of 3,484,996,740 bp from 38,722,186 sequence reads of mycelia, and 3,573,921,960 bp from 39,710,244 high quality sequence reads of sclerotium were obtained. These raw data were assembled into 60,354 contigs and 40,939 singletons, and 56,938 contigs and 37,220 singletons for mycelia and sclerotia, respectively. The transcriptomic data clearly showed that terpenoid biosynthesis was only via the MVA pathwayin *W. cocos*. The production of total triterpenoids and pachymic acid was examined in the dry mycelia and sclerotia. The content of total triterpenoids was 5.36% and 1.43% in mycelia and sclerotia, respectively, and the content of pachymic acid was 0.458% and 0.174%. Some genes involved in the triterpenoid biosynthetic pathway were chosen to be verified by qRT-PCR. The unigenes encoding diphosphomevalonate decarboxylase (Unigene 20430), farnesyl diphosphate synthase (Unigene 14106 and 21656), hydroxymethylglutaryl-CoA reductase (NADPH) (Unigene 6395_All) and lanosterol synthase (Unigene28001_All) were upregulated in the mycelia stage. It is likely that expression of these genes influences the biosynthesis of triterpenoids in the mycelia stage.

## Introduction

*Wolfiporia cocos* Ryvarden et Gilbertson is a saprophytic fungus belonging to the polyporaceae family of the Basidiomycetes, which is usually a parasite in the roots of diverse species of *Pinus*. *W. cocos* is widely distributed in East Asia, Australia, America and Africa [1], [2]. Its dried sclerotia are widely used as a traditional crude drug in China, Korea and Japan, whereas it is used as a kind of food by the native Americans. The dried sclerotium of *W. cocos*, which is known as Fuling in Chinese traditional medicines, is prescribed in many formulations and has been used for several thousand years in China [3], [4]. Fuling has been demonstrated to have spleen-invigorative, stomach-tonifying, sedative, tranquilizing, diuretic, and damp-clearing effects. It is mainly used to treat retention of phlegm and fluid, dysuria, edema, poor appetite with watery stools, palpitations, and insomnia [5]. Recent studies have focused on the cancer-fighting capabilities of Fuling, and clinical trials are still under way to determine the direct effect of *W. cocos*. Polysaccharides and triterpenes are the main active chemical constituents of Fuling, which shows antitumor, anti-inflammatory and antioxidant activities. Many different polysaccharides have been isolated from *W.* cocos [6], [7]. A compound called β-pachyman, which is defined as (1→3)-(1→6)-β-D-glucan, had been isolated by various groups before 1980. Recently, other researchers have isolated and identified different polysaccharides. Almost all of the triterpenes that have been isolated from *W. cocos* can be considered as derivatives of a lanostane skeleton. Pachymic acid (PA), a lanostane-type triterpenoid from *W. cocos*, is a main active compound, which has antitumor and anti-inflammatory activities. PA is widely taken as an index component for evaluation and quality control of Fuling and its formulations.

**Figure 1:**
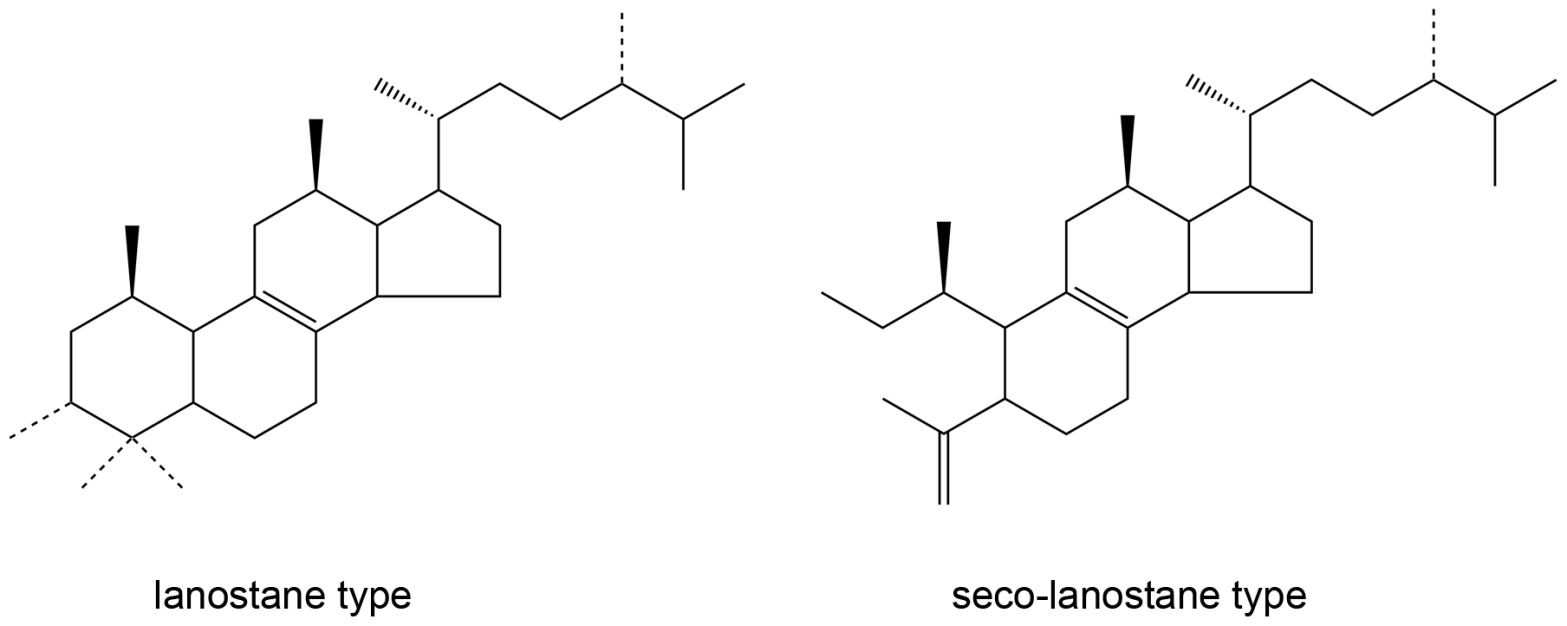
The general structure of lanostane type and seco-lanostane type terpenoids produced by *W. cocos*.

With the application of next-generation sequencing technology, deep-sequencing dependent RNA-Seq, especially the *de novo* sequencing is the most widely used strategy for transcriptomic profiling in non-model organisms [8], [9]. Among the new-generation sequencing methods, 454 pyrosequencing techniques and Illumina sequencing are widely used to analyze transcriptomes. Compared with 454 pyrosequencing, HiSeq 2000 is cost less and much greater output. This makes HiSeq 2000 as an enabling approach for high-throughput counting dependent RNA-seq. *W. cocos* produces tetracyclic lanostanes and other types of triterpenes (Figure 1) [4], [10], [11]. Though there was rare reports on the biosynthesis of triterpenes in *W. cocos,* there were a lots of report on biosynthesis of lanostanes in other organism. The biosynthetic pathway for the backbone of triterpenes from *W. cocos* probably could be proposed after *de novo* RNA-sequencing and terpenoids examiniation.

In this study, we tried to establish a scheme for the common biosynthetic pathway of the triterpenes in *W. cocos* from the transcriptomic data. With the transcriptomic data, we predicted potential genes most likely involved in biosynthesis of triterpenoids, including pachymic acid. This might suggest more triterpene compounds can be figured out from *W. cocos*. The production of triterpenes and pachymic acid and qRT-PCR results indicated that those genes probably involved in terpenoids biosynthesis. Some growth-dependent enzymes could be elucidated and provide a basis for optimizing the cultivation of *W. cocos*. With the development of synthetic biology, this information could promote the application of the triterpene biosynthetic pathway to produce active triterpenoids, and might be helpful for molecular breeding of *W. cocos*.

## Results

### Generation and Assembly of *de novo* Transcriptomic Sequencing Data

To obtain an overview of the *W. cocos* gene expression profile during development, cDNA samples from different developmental stages (mycelium and sclerotium) were prepared and sequenced on an Illumina HiSeq2000 machine. A total of 3,484,996,740 bases from 38,722,186 sequence reads of mycelia, and 3,573,921,960 from 39,710,244 high quality sequence reads of sclerotium were obtained (Table 1).

**Table 1:** Overview of the sequencing and assembly.

Item	Hypha		Sclerotia	
	Sequence (nt)	Bases (bp)	Sequence (nt)	Bases (bp)
Sequencing				
High-quality reads	38,722,186		39,710,244	
Total nucleotide		3,484,996,740		3,573,921,960
assembly				
Number of contigs	60,354		56,938	
Average length of contigs		503		585
N50	765		991	
Total Consensus Sequences	42,037		38,533	
Average length of Unigenes		646		767
N50	814		1037	
Distinct Singletons	40939		37220	

**Figure 2:**
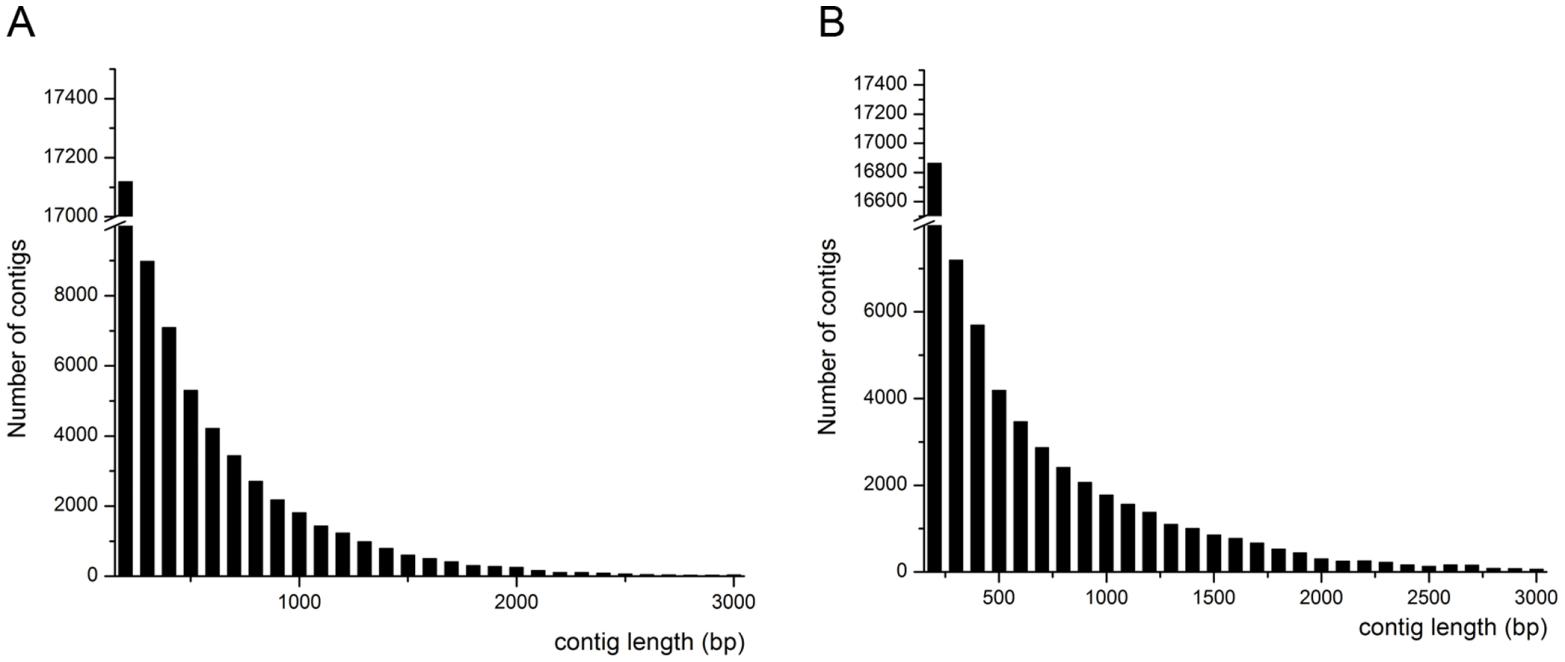
Assembled contig length distribution of *W. cocos* transcriptome. (A) hypha, (B) sclerotium.

These raw data were assembled into 60,354 contigs and 40,939 singletons, and 56,938 contigs and 37,220 singletons for mycelia and sclerotia, respectively. We generated 41,327 unigenes. The mean contig size was 503 or 767 bp with lengths ranging from 200 to 3000 bp (Table 1, Figure 2). The contig size distribution revealed as following: more than half of the contigs (46,501; 81.67%) were between 200 and 1000 bp in length for mycelia and 87.50% (52,808) contigs for sclerotia. The distribution is shown in Figure 2.

To qualify the sequencing and assembling results, 6 contigs (>200 bp) and 6 singletons were randomly selected for RT-PCR analysis. And these RT-PCR products were verified by electrophoresis and Sanger sequencing (data not shown).

### Annotation of Predicted Proteins

BLASTX alignments (e-value cut-off of 10^-4^) between the predicted protein sequences and several protein databases, including GenBank non-redundant and Swiss-Prot, showed that a total of 27,325 (65.90%) predicted proteins could be annotated with known biological functions, whereas the remainder will require more genetic data, which are currently lacking in the database. All the sequencing data was submitted to Genbank. The accession number of this project is PRJNA191862.

**Figure 3:**
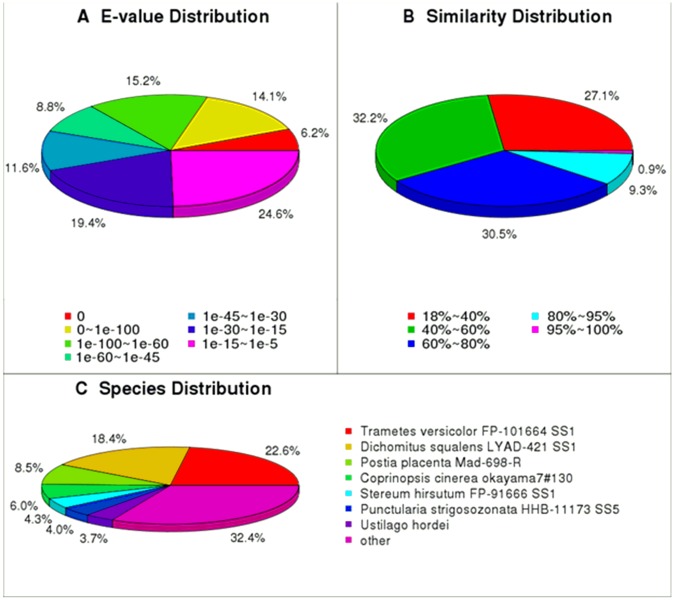
Distribution of the homology search of expressed sequence tags against the nr database.

The identity distribution and species distribution were analyzed (Figure 3). For the identity distribution of the predicted proteins, most of the hits (30.5%) had 60–80% identity with other fungi in the nr database, whereas 10.2% of the sequences had>80% identity (Figure 3). The species distribution of the top BLASTX hits against the nr database for the *W. cocos* transcriptome showed that *W. cocos* genes had the greatest number of matches with genes of *Dichomitus squalens* LYAD-421 SS1 and *Trametes versicolor* FP-101664 SS1. Among these, 22.6% of the unigene sequences had first hits with sequences from *D. squalens* LYAD-421 SS1 and 17% with sequences from *T. versicolor* FP-101664 SS1, followed by other species *Postia placenta* Mad-698-R (8.5%), *Coprinopsis cinerea* okayama 7#130 (6.0%), *Stereum hirsutum* FP-91666 SS1 (4.3%), *Punctularia strigosozonata* HHB-11173 SS5 (4.0%), and *Ustilago hordei* (3.7%). The other 32.4% unigenes had first hits with other fungal species such as *Schizophyllum commune* and *Cryptococcus gattii*.

### Classification of GO and Clusters of Orthologous Groups

**Figure 4:**
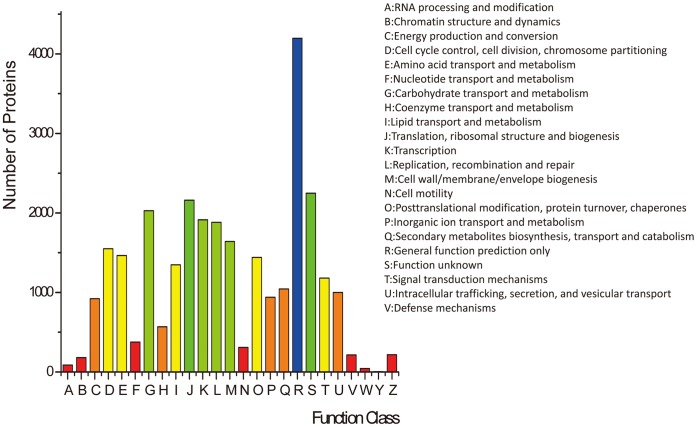
Histogram of COG classification.

GO assignment programs were utilized for functional categorization of annotated genes. In many cases, multiple terms were assigned to the same transcript. These sequences were categorized into 43 main functional groups belonging to three categories, including the biological process, molecular function, and cellular component (Figure 4). Among the biological processes, the dominant GO terms were grouped into either metabolic or cellular processes (Figure 4). Within the molecular function category, there was a high percentage of genes with catalytic activity and binding (Figure 4). For cellular components, those assignments were mostly given to cell components and cell membranes (Figure 4).

**Figure 5:**
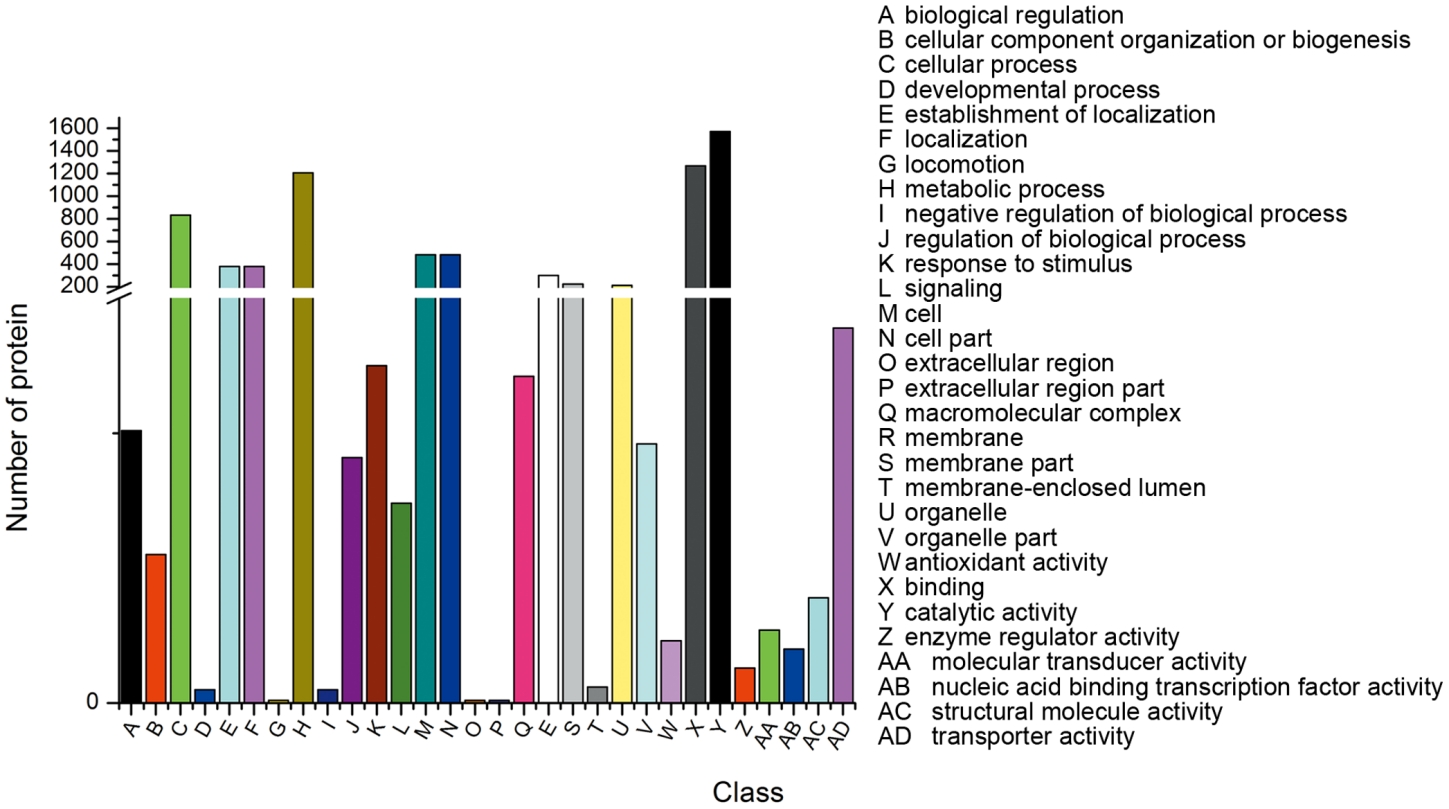
Histogram of GO analysis of transcriptome data.

To evaluate further the completeness of our transcriptomic library and the effectiveness of our annotation process, assignments of clusters of orthologous groups (COGs) were used. Overall, 28,973 proteins were classified as being involved in different processes (Figure 5). Among the 25 COG categories, the majority of the clusters were “General function prediction only” (4196, 14.48%), “Function unknown” (2250, 7.77%), “Translation, ribosomal structure and biogenesis” (2161, 7.46%), “Carbohydrate transport and metabolism” (2028, 7.00%), “Transcription” (1914, 6.61%), and “Replication, recombination and repair” (1882, 6.50%).

### Difference in Gene Expression between Vegetative Mycelia and Sclerotia

**Figure 6:**
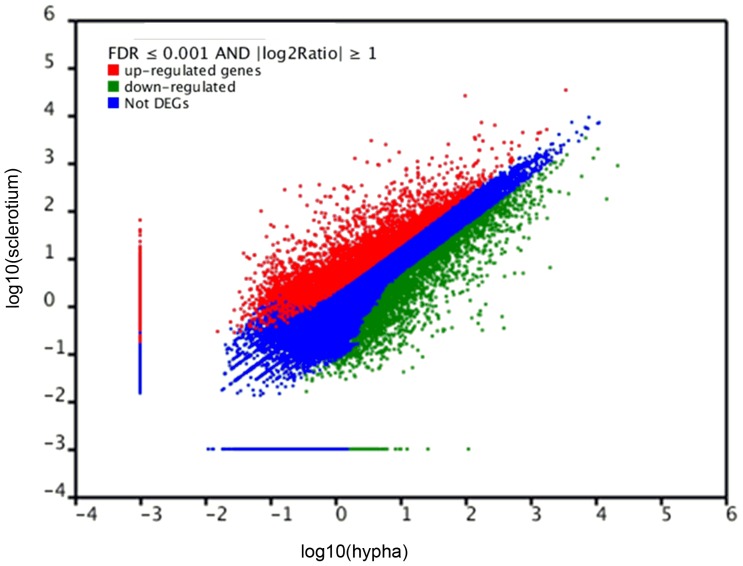
Gene transcription profile between hyphal and sclerotial stages.

The transcriptomic data was analyzed with DEGSeq. Expression of 20,242 genes was upregulated in the sclerotia, and 15,970 in the mycelia (Figure 6). Among the 41,327 unigenes, 5115 showed no significant difference between the hyphal and sclerotial stages.

### Triterpene Synthesis Pathway

The 16,385 unique genes were compared using BLASTX against the KEGG database. These unigenes were assigned to 108 metabolic pathways. The KEGG metabolic pathways that were represented by the *W. cocos* unique genes were metabolic pathways (4576) and biosynthesis of secondary metabolites (2086). Unfortunately, in these KEGG maps of biosynthesis, most enzymes were not mapped to the KEGG pathway database.

**Figure 7:**
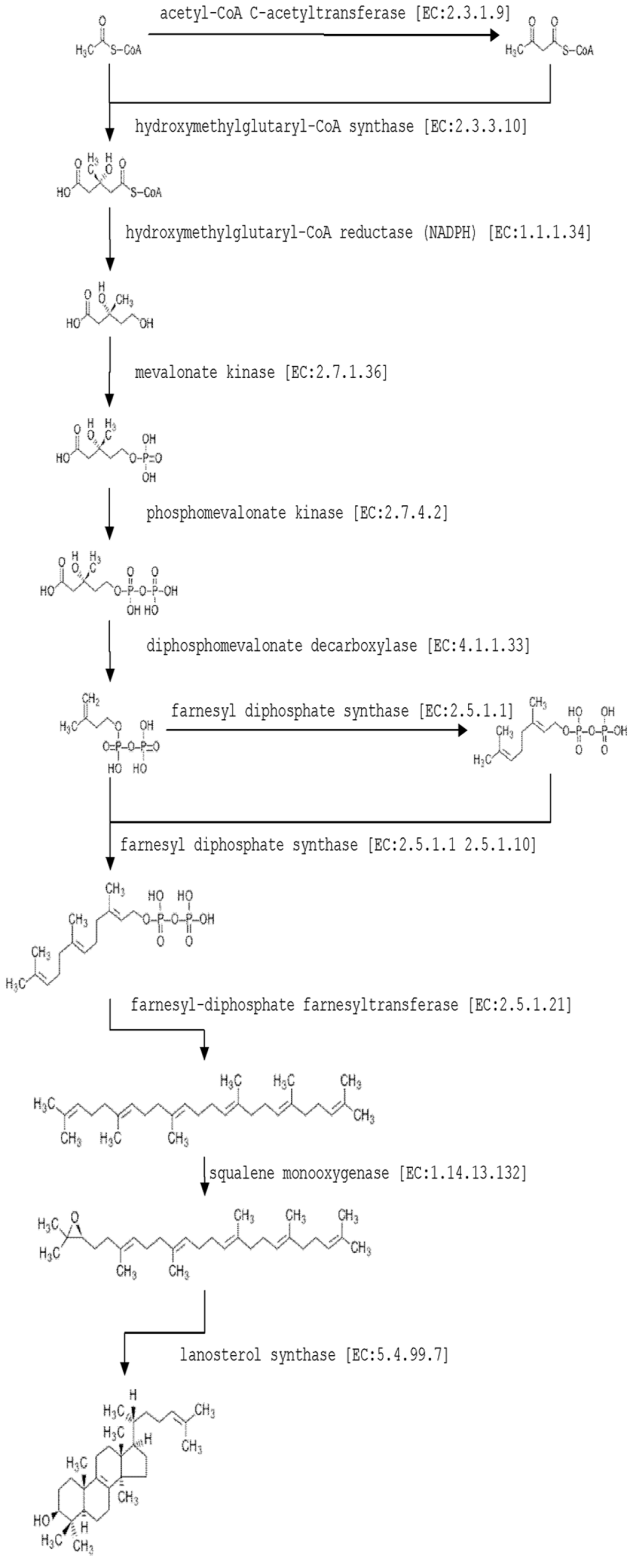
Proposed biosynthetic pathway for triterpene backbone in *W. cocos*.

We focused on 102 and 63 genes assigned to steroid biosynthesis and terpenoid backbone biosynthesis, respectively. It is well known that MVA pathway and MEP pathway are involved in making IPP, the key building block of all isoprenoids, including pigments (chlorophylls and carotenoids), phytohormones (gibberellins), sterols, and other terpenes (Rohmer et al., 1993; Lichtenthaler, 1999). Only those genes involved in the MVA (mevalonate) pathway can be found in the transcriptomic data of *W. cocos*. We proposed that terpenoid biosynthesis of *W. cocos* was pobably via the MVA pathway not the MEP pathway. In the KEGG map of terpenoid backbone and steroid biosynthesis, key enzymes (such as farnesyl diphosphate synthase and geranylgeranyl diphosphate synthase) can be refered to unique genes in the annotated transcriptomic data. We tried here to assign these genes into the biosynthetic pathway of the lanostane backbone (Figure 7 and Table 2).

**Table 2:** Possible Genes encode enzymes involved in triterpenoid biosynthetic pathway.

Enzyme	Unigenes
acetyl-CoA C-acetyltransferase [EC:2.3.1.9]	Unigene1133_All, Unigene16352_All, Unigene1134_All
hydroxymethylglutaryl-CoA synthase [EC:2.3.3.10]	Unigene7232_All, Unigene16882_All
hydroxymethylglutaryl-CoA reductase (NADPH) [EC:1.1.1.34]	Unigene14752_All, Unigene14229_All, Unigene1615_All, Unigene6395_All
mevalonate kinase [EC:2.7.1.36]	Unigene8508_All, Unigene11355_All
phosphomevalonate kinase [EC:2.7.4.2]	
diphosphomevalonate decarboxylase [EC:4.1.1.33]	Unigene20430_All, Unigene26804_All, Unigene26802_All
farnesyl diphosphate synthase [EC:2.5.1.1 2.5.1.10]	Unigene24144_All, Unigene 14106_All, Unigene 21656_All
farnesyl-diphosphate farnesyltransferase [EC:2.5.1.21]	Unigene19472_All,Unigene36832_All,Unigene16754_All
squalene monooxygenase [EC:1.14.13.132]	Unigene11783_All
lanosterol synthase [EC:5.4.99.7]	Unigene21626_All, Unigene28001_All
7-O-acetyltransferase	Unigene12366_All
cytochrome P450, family 51 (sterol 14-demethylase)[EC:1.14.13.70]	Unigene33807_All, Unigene33454_All, Unigene11573_All, Unigene11556_All, Unigene11555_All, Unigene16152_All, Unigene1229_All, Unigene3467_All, Unigene1230_All, Unigene9503_All, Unigene12189_All, Unigene34077_All, Unigene19892_All, Unigene3634_All, Unigene8630_All, Unigene12003_All, Unigene27534_All, Unigene9706_All, Unigene4106_All
delta14-sterol reductase [EC:1.3.1.70]	Unigene17860_All,Unigene22450_All
methylsterol monooxygenase [EC:1.14.13.72]	Unigene16802_All
sterol-4alpha-carboxylate 3-dehydrogenase (decarboxylating) [EC:1.1.1.170]	Unigene38744_All, Unigene36928_All, Unigene38029_All, Unigene19740_All, Unigene10948_All, Unigene14000_All, Unigene16011_All, Unigene29933_All
3-keto steroid reductase [EC:1.1.1.270]	Unigene35957_All, Unigene4322_All, Unigene6340_All
cholestenol delta-isomerase [EC:5.3.3.5]	Unigene11128_All, Unigene16177_All
sterol 24-C-methyltransferase [EC:2.1.1.41]	Unigene37920_All, Unigene7536_All, Unigene34791_All, Unigene38291_All, Unigene11148_All
sterol 24-C-methyltransferase [EC:2.1.1.41]	Unigene11128_All, Unigene16177_All
delta24(24(1))-sterol reductase [EC:1.3.1.71]	Unigene21541_All, Unigene9213_All

### Triterpene Production

The production of total triterpenoids and pachymic acid was analyzed in the dry mycelia and sclerotia. More terpenoid was produced in the mycelia. The content of total triterpenoid was 5.36% and 1.43% in mycelia and sclerotia, respectively, and the content of pachymic acid was 0.458% and 0.174%.

**Figure 8:**
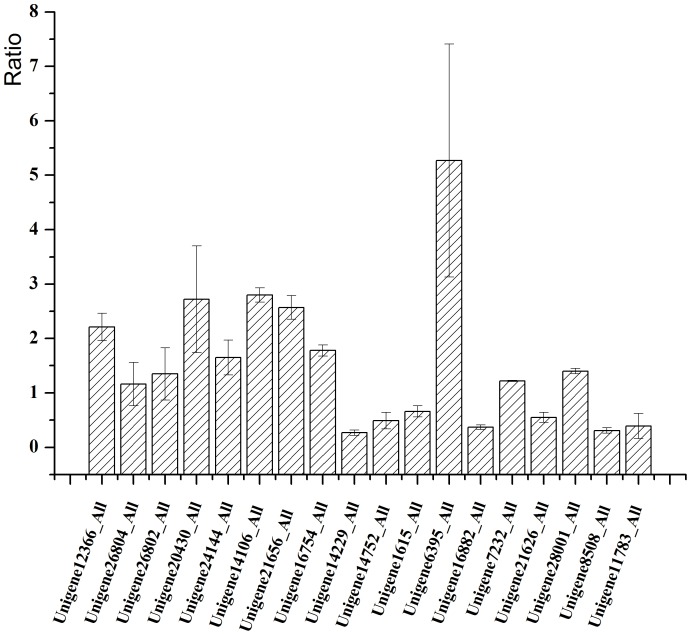
Histogram of qRT-PCR data of genes involved in biosynthetic pathway of terpenoids.

The transcriptomic data showed that some genes involved in the triterpenoid biosynthetic pathway were upregulated in the hyphal stage. Eighteen of these genes were selected for verification by qRT-PCR. The expression pattern of these genes is shown in Figure 8. The unigenes encoding diphosphomevalonate decarboxylase (unigene 20430), farnesyl diphosphate synthase (unigenes 14106 and 21656), hydroxymethylglutaryl-CoA reductase (NADPH) (unigene 6395_All), and lanosterol synthase (unigene 28001_All) were upregulated in the hyphal stage. This showed their commitment to triterpenoid production, and it is likely that these genes were involved in triterpenoid biosynthesis in the hyphal stage.

In the structure of pachymic acid, an O-acetyl group was introduced into the 3-C of the lanosterol backbone. Analysis of the possible O-acetyltransferase involved in this reaction suggested that unigene 12366_All probably encoded a salutaridinol 7-O-acetyltransferase. This enzyme reaction in the KEGG reaction database showed introduction of an acetyl group at a similar site. qRT-PCR and transcriptomic data of unigene 12366_All also showed upregulation in the mycelia stage. This was also consistent with the pachymic acid higher production in the mycelia. We proposed that unigene 12366_all is probably involved in the biosynthesis of pachymic acid.

## Discussion

We propose a biosynthetic pathway for pachymic acid on the basis of our transcriptomic data. The production of terpenoid products was also examined. qRT-PCR showed that the transcriptomic data of high-throughput sequencing was reliable for analysis of the transcriptomic pattern of fungi. qRT-PCR can also help to verify the genes involved in the biosynthetic pathway, by transcription pattern analysis. In the KEGG pathway database, triterpenoid biosynthesis needs phosphomevalonate kinase. However, in the transcriptomic data, no gene encoding this enzyme could be found. Orthologous blast results showed that unigene 8508_All and 11355_All both encoded mevalonate kinase. It could be that one of these two genes is involved in the transformation of phosphomevalonate to diphosphomevalonate.

P450 gene and glycosyl transferase are also needed for triterpenoid biosynthesis. The transcriptome of *W. cocos* showed that there was at least 249 putative cytochrome P450s that can be detected with transcription (Supplementary Table S1). Among these, unigenes 34077_All, 16152_All and 12189_All encoded putative lanosterol 14-α demethylase. These genes probably formed 14-demethyl lanosterol derivatives.

From the genes that may be involved in the terpenoids biosynthetic pathway, it could be possible to isolate new triterpenoid products from *W. cocos*. Our results showed that more triterpenoids were produced in the hyphal stage than in the sclerotium. This suggests that modern fermentation methods could be introduced for production of triterpenoid products from *W. cocos*.

We still cannot find any reports about the biosynthetic pathway for triterpenoids in *W. cocos*. Our present results provide a basis for further research. With the development of synthetic biology, the biosynthetic pathway for pachymic acid can be assembled in other fungi that can be easily manipulated genetically and used to increase the production of pachymic acid. This will probably promote research on *W. cocos*.

We showed that this type of *de novo* sequencing can be used to elucidate biosynthetic pathways for production of natural bioactive products. This gives a new incentive to analyze more medicinally important fungi.

## Materials and Methods

### Media and Culture Condition

Sclerotia of *W. cocos* were collected from the field in Yingshan county, Hubei province, China in 2012, and it was approved by the Science and Technology Bureau of Yingshan county. Mycelia of *W. cocos* were obtained from outgrowth of sclerotia on potato dextrose agar plates at 28°C.

### RNA Isolation and Sequencing

The sclerotia and mycelia of *W. cocos* were immediately stored in liquid nitrogen until further processing. Total RNA was extracted from1 g sclerotia and mycelia using TRIzol reagent (Life Technologies Inc., Carlsbad CA, USA) and was treated with RNase-free DNase I for 30 min at 37°C (Qiagen Inc., Duesseldorf, Germany) to remove residual DNA. The RNA sample was sent to BGI (Beijing Genomic Institute) for RNA sequencing(BGI Inc., Shenzhen,China). Sequencing was carried out as follows. Beads with oligo(dT) were used to isolate poly(A) mRNA from total RNA. Fragmentation buffer was added to fragment mRNA into short fragments of 200–700 bp. Taking these short fragments as templates, random hexamer primer was used to synthesize the first-strand cDNA. The second-strand cDNA was synthesized using buffer, dNTPs, RNaseH and DNA polymerase I. Short fragments were purified with QiaQuick PCR extraction kit and resolved with EB buffer for end reparation and adding poly(A) (Qiagen Inc., Duesseldorf, Germany). The short fragments were connected with sequencing adapters, and after agarose gel electrophoresis, suitable fragments were selected for PCR amplification as templates. Finally, the library was sequenced using Illumina HiSeq 2000(Illumina Inc., San Diego CA, USA).

### Sequence Assembling and Analysis

Raw reads produced from sequencing machines contain dirty reads with adapters or unknown or low quality bases. These data negatively affect bioinformatics analysis. Therefore, dirty raw reads were discarded. Transcriptome *de novo* assembly was carried out with a short read assembling program: Trinity [12]. Trinity combined reads with a certain length of overlap to form longer fragments without N, which were called contigs. These contigs were subjected to further processing of sequence clustering to form longer sequences without N. Such sequences were defined as unigenes. When multiple samples from the same species were sequenced, unigenes from each sample’s assembly were taken for further processing of sequence splicing and redundancy removal to acquire nonredundant unigenes, as long as possible. In the final step, BLASTX alignment (e value<0.00001) between unigenes and protein databases such as nr, Swiss-Prot, KEGG and COG, was performed, and the best alignments were used to decide upon the sequence direction of the unigenes. If the results of different databases conflicted with each other, a priority order of nr, Swiss-Prot, KEGG and COG was followed when deciding the sequence direction of the unigenes. When a unigene was not aligned to any of the above databases, ESTScan software was used to decide upon the sequence direction. For unigenes with sequence directions, we provided their sequences from the 5′ to 3′ end; for those without any direction, we provided their sequences from assembly software. The length of sequences assembled was a criterion for assembly success. The distribution of the lengths of contigs, scaffolds, and unigenes was calculated.

### Unigene Function Annotation

Unigene sequences were first aligned by BLASTX to protein databases such as nr, Swiss-Prot, KEGG and COG (e-value<0.00001), retrieving proteins with the highest sequence similarity with the given unigenes, along with their protein functional annotations [13]. The results were included in the folder annotation.

The KEGG database contains systematic analysis of inner-cell metabolic pathways and functions of gene products. It helps when studying complicated biological behavior of genes. With KEGG annotation, we obtained the pathway annotation of the unigenes.

The COG is a database in which orthologous gene products are classified. Every protein in COG is assumed to have evolved from an ancestor protein, and the whole database is built on coding proteins with complete genome as well as system evolution relationships of bacteria, algae and eukaryotic creatures. Unigenes were aligned to the COG database to predict and classify possible functions of the unigenes.

KEGG is a database that is able to analyze gene products during metabolic processes and related gene functions in the cellular processes. With the help of the KEGG database, we studied the biological complex behavior of genes, and by KEGG annotation we obtained the pathway annotation for the unigenes.

Unigenes were first aligned by BLASTX (e-value<0.00001) to protein databases in the priority order of nr, Swiss-Prot, KEGG and COG. Unigenes aligned to a higher priority database were not aligned to a lower priority database. Proteins with the highest rank in the BLASTX results were used to decide the coding region sequences of the unigenes, and then the coding region sequences were translated into amino sequences with the standard codon table. The nucleotide (5′–3′) and amino acid sequences of the unigene coding regions were acquired. Unigenes that could not be aligned to any database were scanned by ESTScan, producing a nucleotide sequence (5′–3′) and amino sequence of the predicted coding region.

### Unigene Expression Difference Analysis

The calculation of unigene expression used the RPKM method (Reads per kb per Million reads) [14], with the following formula:
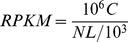



RPKM was the expression of unigene A, and C the number of reads that were uniquely aligned to unigene A; N the total number of reads that were uniquely aligned to all unigenes; and L the base number in the CDS (Coding sequence) of unigene A. The RPKM method was able to eliminate the influence of different gene lengths and sequencing levels on the calculation of gene expression. Therefore, the calculated gene expression could be directly used to compare the difference in gene expression between the samples.

False discovery rate (FDR) control is a statistical method used in multiple hypothesis testing to correct for p values. In practical terms, the FDR is the expected false discovery rate; for example, if 1000 observations were experimentally predicted to be different, and a maximum FDR for these observation was 0.1, then 100 out of these observations would be expected to be false discovered [15]. When we obtained FDR, we used the ratio of RPKMs of the two samples at the same time. The smaller the FDR and the larger the ratio, the larger was the difference in the expression level between the two samples. In our analysis, we chose those with FDR≤0.001 and ratio>2. DEGs (differentially expressed genes) were then carried out into gene ontology (GO) functional analysis and KEGG pathway analysis. The DEGs was analyzed with DEGSeq [16].

### Quantification of the Total Triterpenoids by Colorimetry

The content of the total triterpenoids from *W. cocos* was determined by the following colorimetric method [17]. An aliquot of the dried powder of the sclerotia or mycelia of *W. cocos* (60 mesh, 1.0 g) was extracted for 1 h with 50 ml acetone under sonication at 30°C. Five milliliters of extracts was centrifuged at 2500 rpm for 10 min. One milliliter of supernatant was evaporated to dryness in an 80°C water bath, and 0.4 ml 5% vanillin/glacial acetic acid (w/v) and 1 ml 70% perchloric acid solution were added successively to the tube. The solution was heated for 15 min at 60°C and then cooled in an ice-water bath to the ambient temperature. The absorbance of the sample was measured at 548 nm after addition of 5 ml glacial acetic acid, with ursolic acid used as the standard.

### Quantification of Pachymic Acid

Two grams of powder of dried sclerotia and mycelia was weighed, extracted with 20 ml methanol for 20 min under sonication at 42°C, and then centrifuged at 3000 rpm for 10 min. The residue was similarly extracted by the same method. Both supernatants were combined and made up to 50 ml with methanol. Pachymic acid were measured by HPLC. The HPLC system consisted of an ODS-80TM column (1504.6 mm i.d.) at 40°C and an injection volume of 20 µl. The mobile phase was acetonitrile, water and acetic acid (800∶200:1). The flow rate was 1.0 ml/min. The peaks were detected at 210 nm for pachymic acid. The content of pachymic acid was calculated on the basis of the dry weight of the sclerotia and mycelia.

### qRT-PCR Confirmation of Expression of Genes Probably Involved in Biosynthesis of Pachymic Acid

Real-time PCR was used to verify the expression of genes probably involved in pachymic acid biosynthesis. The primers for real-time PCR are listed in Table 3. SYBR Green real-time PCR Master Mix Plus Kit (Toyobo Inc., Osaka Japan) was used to carry out qRT-PCR according to the manufacturer’s instructions with a real-time thermal cycler (ABI7500; Life Technologies Inc., Carlsbad CA, USA). The PCR conditions were as follows: denaturation at 94°C for 2 min; 40 rounds of 94°C for 15 s, 65°C for 15 s and 72°C for 45 s, with a final step of 72°C for 10 min. The ReverTra Ace kit (Toyobo Inc., Osaka Japan) was used to obtain the first-strand cDNA from 2 µg total RNA with oligo(dT)20. All quantitative PCRs were repeated 3 times with three technical replications per experiments. The gene transcription level in mycelia and sclerotia was compared with the results of transcriptomic analysis.

**Table 3:** Primers for qRT-PCR to verify possible genes involved in the biosynthesis of triterpenoid.

Target CDSs	Primer	sequence(5′ to 3′)
Unigene7232_All	1-F	CCTCGCGATGGAGATGTATTT
	1-R	CCCAGTCCGATGGTGTATTT
Unigene16882_All	2-F	GCAAGTACCGTCGCAAGTA
	2-R	TATGCGAGAGCAAGGAAGATG
Unigene14752_All	3-F	CCTGCGATTGACTTCCCTAC
	3-R	CGAACGCCTCCTTGACAATA
Unigene14229_All	4-F	GTTCGCGTCATCCTGTACTT
	4-R	GCGTCGACTATGGTACGTATTT
Unigene1615_All	5-F	CTACTGTGCGGTTCTGTCTATC
	5-R	GTGTCCAGCTTGTACGACTT
Unigene6395_All	6-F	CGGGAATGGAGCAACAATAAC
	6-R	TCCTACTCCGGCGAGATAAA
Unigene8508_All	7-F	GCTCGTCCCTGACGATTTC
	7-R	CCAAGCCAGACCCTCCTA
Unigene20430_All	8-F	CGAGCCGTACGATTTGAGAT
	8-R	GACATAGTGGGCAAGGAGATAG
Unigene26804_All	9-F	GTTCACGGATGGCAAGAATAAAG
	9-R	CGTTGGACCGGTATTGATGA
Unigene26802_All	10-F	CCCTTATCTTCTTGCCCACTATG
	10-R	TCAACTTGGTATCCCGCTTTC
Unigene24144_All	11-F	CACAGGCGGTTACAACACTA
	11-R	CGCTATCTATTTCGCAGGAGAG
Unigene14106_All	12-F	GGGTGCGTGGAAGCATATTA
	12-R	GCGGAAGTGGGAGTCTTTAC
Unigene21656_All	13-F	CGTCCGACTGGTCAACAAA
	13-R	GACCATCAACTCGGCTAACTAC
Unigene16754_All	14-F	GCCATGTTCTGATGCTACCT
	14-R	CGAGGGACGTGAACGATAAA
Unigene11783_All	15-F	CCGTCCCTGCGTAAACATAA
	15-R	TCGCTGCTCTCTGGAATTG
Unigene21626_All	16-F	GAGGTCTTTGGTGCCATCAT
	16-R	CCGCCCGATAGTTTGGATAAG
Unigene28001_All	17-F	GATTCACACTCGGACCGTTTA
	17-R	CAGTCGATGCTGTAGTAGTCTTG
Unigene12366_All	18-F	CGAAGTGTGCGAGAGTATGT
	18-R	CCACGACCTTATCGAGCATATC
Unigene9842_All(tubulin alpha)	19-F	ACTCCAGCTTGGACTTCTTG
	19-R	TCTTCGTCTTCCACTCCTTTG

## Supporting Information

Table S1 **The putative P450 encoding genes in** ***Wolfiporia cocos.***
(DOCX)
