# Supplementary material for: De Novo Sequencing and Transcriptome Analysis of Wolfiporia cocos to Reveal Genes Related to Biosynthesis of Triterpenoids
Source: PLoS One. 2013 Aug 14;8(8):e71350. doi: 10.1371/journal.pone.0071350 (PMC3743799; doi:10.1371/journal.pone.0071350)
Supplement: Table S1 — The putative P450 encoding genes in Wolfiporia cocos. (DOCX) [file pone.0071350.s001.docx]

Table S1 The putative P450 encoding genes in Wolfiporia cocos

| **GeneID** | **Rawreads** | | **Nr-annotation** |
| --- | --- | --- | --- |
|  | **Hyphae** | **Sclerotium** |  |
| Unigene5927_All | 18 | 2 | cytochrome P450 [Auricularia delicata TFB-10046 SS5] |
| Unigene34455_All | 3 | 37 | cytochrome P450 [Postia placenta] |
| Unigene15443_All | 26 | 70 | cytochrome P450 [Postia placenta] |
| Unigene36364_All | 5 | 13 | cytochrome P450 [Postia placenta] |
| Unigene39504_All | 1 | 10 | cytochrome P450 [Trametes versicolor FP-101664 SS1] |
| Unigene40426_All | 1 | 4 | cytochrome P450 98A3 [Dichomitus squalens LYAD-421 SS1] |
| Unigene35192_All | 48 | 175 | cytochrome P450 [Postia placenta] |
| Unigene30656_All | 3 | 5 | cytochrome P450 [Postia placenta] |
| Unigene8144_All | 14 | 15 | cytochrome P450 [Trametes versicolor FP-101664 SS1] |
| Unigene21054_All | 8 | 5 | cytochrome P450 [Postia placenta] |
| Unigene8710_All | 283 | 97 | cytochrome P450 [Postia placenta] |
| Unigene16262_All | 4 | 10 | cytochrome P450 [Postia placenta] |
| Unigene16261_All | 24 | 23 | cytochrome P450 [Postia placenta] |
| Unigene9503_All | 22 | 91 | cytochrome P450 [Postia placenta] |
| Unigene2381_All | 21 | 803 | cytochrome P450 [Postia placenta] |
| Unigene36323_All | 15 | 120 | cytochrome P450 [Postia placenta] |
| Unigene577_All | 14 | 22 | cytochrome P450 [Postia placenta] |
| Unigene578_All | 90 | 412 | cytochrome P450 [Postia placenta] |
| Unigene1690_All | 999 | 728 | cytochrome P450 [Postia placenta] |
| Unigene22127_All | 177 | 161 | cytochrome P450 [Postia placenta] |
| Unigene23770_All | 99 | 80 | cytochrome P450 [Dichomitus squalens LYAD-421 SS1] |
| Unigene20374_All | 164 | 120 | cytochrome P450 [Punctularia strigosozonata HHB-11173 SS5] |
| Unigene36916_All | 2 | 4 | cytochrome P450 [Punctularia strigosozonata HHB-11173 SS5] |
| Unigene12003_All | 22 | 0 | cytochrome P450 [Postia placenta] |
| Unigene33454_All | 0 | 18 | cytochrome P450 [Trametes versicolor FP-101664 SS1] |
| Unigene30784_All | 26 | 34 | cytochrome P450 [Postia placenta] |
| Unigene1293_All | 12 | 55 | cytochrome P450 [Postia placenta] |
| Unigene1658_All | 96 | 364 | cytochrome P450 [Postia placenta] |
| Unigene38570_All | 3 | 26 | cytochrome P450 [Postia placenta] |
| Unigene10656_All | 291 | 1582 | cytochrome P450 [Postia placenta] |
| Unigene2512_All | 193 | 147 | cytochrome P450 [Postia placenta] |
| Unigene17468_All | 26 | 43 | cytochrome P450 [Punctularia strigosozonata HHB-11173 SS5] |
| Unigene6090_All | 882 | 1718 | cytochrome P450 [Postia placenta] |
| Unigene6556_All | 245 | 279 | cytochrome P450 [Postia placenta] |
| Unigene21460_All | 935 | 2884 | cytochrome P450 [Stereum hirsutum FP-91666 SS1] |
| Unigene10657_All | 85 | 605 | cytochrome P450 [Postia placenta] |
| Unigene19012_All | 758 | 472 | cytochrome P450 [Trametes versicolor FP-101664 SS1] |
| Unigene35827_All | 4 | 25 | cytochrome P450 [Postia placenta] |
| Unigene29385_All | 61 | 15 | cytochrome P450 [Postia placenta] |
| Unigene22150_All | 26 | 28 | cytochrome P450 [Stereum hirsutum FP-91666 SS1] |
| Unigene40853_All | 0 | 7 | cytochrome P450 [Stereum hirsutum FP-91666 SS1] |
| Unigene31616_All | 14 | 1 | cytochrome P450 [Stereum hirsutum FP-91666 SS1] |
| Unigene1702_All | 1618 | 2687 | cytochrome P450 [Postia placenta] |
| Unigene22641_All | 95 | 104 | cytochrome P450 [Postia placenta] |
| Unigene21131_All | 627 | 274 | cytochrome P450 [Postia placenta] |
| Unigene14640_All | 170 | 141 | cytochrome P450 [Postia placenta] |
| Unigene10068_All | 80 | 69 | cytochrome P450 [Postia placenta] |
| Unigene5575_All | 119 | 118 | cytochrome P450 [Postia placenta] |
| Unigene3298_All | 30 | 60 | cytochrome P450 [Postia placenta] |
| Unigene20602_All | 402 | 18184 | cytochrome P450 [Postia placenta] |
| Unigene21130_All | 58 | 7 | cytochrome P450 [Postia placenta] |
| Unigene31983_All | 18 | 3 | cytochrome P450 [Postia placenta] |
| Unigene24424_All | 263 | 216 | cytochrome P450 [Postia placenta] |
| Unigene6695_All | 1878 | 2357 | cytochrome P450 [Postia placenta] |
| Unigene18014_All | 265 | 713 | cytochrome P450 [Postia placenta] |
| Unigene19660_All | 335 | 834 | cytochrome P450 [Postia placenta] |
| Unigene3553_All | 565 | 1396 | cytochrome P450 [Postia placenta] |
| Unigene20966_All | 194 | 342 | cytochrome P450 [Postia placenta] |
| Unigene478_All | 2330 | 3202 | cytochrome P450 [Postia placenta] |
| Unigene17753_All | 34 | 36 | cytochrome P450 [Postia placenta] |
| Unigene34142_All | 2 | 10 | cytochrome P450 [Postia placenta] |
| Unigene10936_All | 2 | 28 | cytochrome P450 [Postia placenta] |
| Unigene13085_All | 89 | 1149 | cytochrome P450 [Postia placenta] |
| Unigene37066_All | 0 | 125 | cytochrome P450 [Postia placenta] |
| Unigene12068_All | 708 | 421 | cytochrome P450 [Postia placenta] |
| Unigene11225_All | 191 | 135 | cytochrome P450 [Dichomitus squalens LYAD-421 SS1] |
| Unigene5731_All | 2045 | 1694 | cytochrome P450 [Postia placenta] |
| Unigene2371_All | 7349 | 30 | cytochrome P450 [Postia placenta] |
| Unigene36479_All | 6 | 117 | cytochrome P450 [Postia placenta] |
| Unigene34249_All | 1 | 1434 | cytochrome P450 [Postia placenta] |
| Unigene35341_All | 11 | 19 | cytochrome P450 [Trametes versicolor FP-101664 SS1] |
| Unigene14552_All | 1070 | 73 | cytochrome P450 [Trametes versicolor FP-101664 SS1] |
| Unigene17663_All | 6 | 2 | cytochrome P450 [Postia placenta] |
| Unigene34260_All | 0 | 94 | cytochrome P450 [Postia placenta] |
| Unigene28748_All | 89 | 10 | cytochrome P450 [Postia placenta] |
| Unigene17696_All | 48 | 51 | cytochrome P450 [Postia placenta] |
| Unigene37510_All | 8 | 16 | cytochrome P450 [Postia placenta] |
| Unigene30976_All | 4 | 5 | cytochrome P450 [Postia placenta] |
| Unigene36938_All | 0 | 37 | cytochrome P450 [Postia placenta] |
| Unigene36939_All | 4 | 68 | cytochrome P450 [Postia placenta] |
| Unigene3499_All | 114 | 400 | cytochrome P450 [Postia placenta] |
| Unigene7048_All | 131 | 464 | cytochrome P450 [Stereum hirsutum FP-91666 SS1] |
| Unigene30484_All | 18 | 2 | cytochrome P450 [Postia placenta] |
| Unigene27449_All | 5 | 4 | cytochrome P450 [Postia placenta] |
| Unigene27450_All | 9 | 0 | cytochrome P450 [Postia placenta] |
| Unigene36360_All | 1 | 2 | cytochrome P450 [Dichomitus squalens LYAD-421 SS1] |
| Unigene36359_All | 0 | 8 | cytochrome P450 [Dichomitus squalens LYAD-421 SS1] |
| Unigene12004_All | 33 | 129 | cytochrome P450 [Dichomitus squalens LYAD-421 SS1] |
| Unigene19714_All | 21 | 52 | cytochrome P450 [Postia placenta] |
| Unigene17594_All | 44 | 10 | cytochrome P450 [Postia placenta] |
| Unigene2036_All | 264 | 129 | cytochrome P450 [Postia placenta] |
| Unigene26245_All | 39 | 2 | cytochrome P450 [Postia placenta] |
| Unigene15383_All | 91 | 148 | cytochrome P450 [Postia placenta] |
| Unigene6103_All | 364 | 504 | cytochrome P450 [Postia placenta] |
| Unigene19892_All | 10 | 37 | cytochrome P450 [Postia placenta] |
| Unigene16434_All | 4 | 1 | cytochrome P450 [Postia placenta] |
| Unigene16435_All | 12 | 20 | cytochrome P450 [Postia placenta] |
| Unigene30169_All | 1 | 0 | cytochrome P450 [Postia placenta] |
| Unigene20375_All | 42 | 25 | cytochrome P450 [Punctularia strigosozonata HHB-11173 SS5] |
| Unigene1954_All | 558 | 407 | cytochrome P450 [Postia placenta] |
| Unigene15235_All | 65 | 68 | cytochrome P450 [Postia placenta] |
| Unigene15236_All | 210 | 155 | cytochrome P450 [Postia placenta] |
| Unigene16003_All | 405 | 377 | cytochrome P450 [Stereum hirsutum FP-91666 SS1] |
| Unigene6302_All | 691 | 2319 | cytochrome P450 [Trametes versicolor FP-101664 SS1] |
| Unigene33807_All | 7 | 8311 | cytochrome P450 [Postia placenta] |
| Unigene3634_All | 1231 | 2975 | cytochrome P450 [Postia placenta] |
| Unigene33806_All | 5 | 8752 | cytochrome P450 [Postia placenta] |
| Unigene8630_All | 919 | 2110 | cytochrome P450 [Postia placenta] |
| Unigene33897_All | 1007 | 722 | cytochrome P450 [Postia placenta] |
| Unigene22515_All | 341 | 880 | cytochrome P450 [Postia placenta] |
| Unigene17128_All | 10071 | 16210 | cytochrome P450 [Postia placenta] |
| Unigene8720_All | 748 | 1759 | cytochrome P450 [Postia placenta] |
| Unigene4106_All | 1157 | 285 | cytochrome P450 [Postia placenta] |
| Unigene5790_All | 1 | 7 | cytochrome P450 [Taiwanofungus camphoratus] |
| Unigene6164_All | 1045 | 12172 | cytochrome P450 [Postia placenta] |
| Unigene1229_All | 14 | 104 | cytochrome P450 [Postia placenta] |
| Unigene1230_All | 85 | 362 | cytochrome P450 [Postia placenta] |
| Unigene1511_All | 20748 | 17821 | cytochrome P450 [Postia placenta] |
| Unigene35680_All | 9 | 488 | cytochrome P450 [Taiwanofungus camphoratus] |
| Unigene19721_All | 599 | 51 | cytochrome P450 [Dichomitus squalens LYAD-421 SS1] |
| Unigene19637_All | 26 | 1869 | cytochrome P450 [Postia placenta] |
| Unigene19907_All | 152 | 148 | cytochrome P450 [Postia placenta] |
| Unigene1271_All | 26 | 41 | cytochrome P450 [Postia placenta] |
| Unigene1270_All | 0 | 8 | cytochrome P450 [Postia placenta] |
| Unigene22095_All | 79 | 143 | cytochrome P450 [Postia placenta] |
| Unigene26757_All | 44 | 59 | cytochrome P450 [Postia placenta] |
| Unigene19144_All | 82 | 82 | cytochrome P450 [Postia placenta] |
| Unigene21071_All | 2862 | 4780 | cytochrome P450 [Postia placenta] |
| Unigene16964_All | 56 | 4697 | cytochrome P450 [Postia placenta] |
| Unigene40875_All | 0 | 6 | cytochrome P450 [Postia placenta] |
| Unigene38839_All | 18 | 18 | cytochrome P450 [Postia placenta] |
| Unigene6646_All | 393 | 531 | cytochrome P450 [Postia placenta] |
| Unigene34077_All | 34 | 125 | cytochrome P450 [Postia placenta] |
| Unigene16152_All | 421 | 5691 | cytochrome P450 [Punctularia strigosozonata HHB-11173 SS5] |
| Unigene12189_All | 1069 | 4658 | cytochrome P450 [Postia placenta] |
| Unigene1846_All | 49 | 458 | cytochrome P450 [Postia placenta] |
| Unigene10614_All | 3 | 30 | cytochrome P450 [Postia placenta] |
| Unigene10613_All | 15 | 47 | cytochrome P450 [Postia placenta] |
| Unigene3467_All | 8 | 39 | cytochrome P450 [Postia placenta] |
| Unigene8651_All | 202 | 665 | cytochrome P450 [Postia placenta] |
| Unigene2748_All | 469 | 1563 | cytochrome P450 [Postia placenta] |
| Unigene1478_All | 534 | 280 | cytochrome P450 [Postia placenta] |
| Unigene21307_All | 0 | 10 | cytochrome P450 [Trametes versicolor FP-101664 SS1] |
| Unigene21308_All | 14 | 45 | cytochrome P450 [Trametes versicolor FP-101664 SS1] |
| Unigene29863_All | 4 | 0 | cytochrome P450 [Dichomitus squalens LYAD-421 SS1] |
| Unigene40152_All | 1 | 7 | cytochrome P450 [Stereum hirsutum FP-91666 SS1] |
| Unigene7750_All | 19 | 86 | cytochrome P450 [Postia placenta] |
| Unigene12621_All | 12 | 2362 | cytochrome P450 [Postia placenta] |
| Unigene24825_All | 69 | 123 | cytochrome P450 [Postia placenta] |
| Unigene17913_All | 18 | 77 | cytochrome P450 [Postia placenta] |
| Unigene31596_All | 17 | 13 | cytochrome P450 [Dichomitus squalens LYAD-421 SS1] |
| Unigene36478_All | 6 | 318 | cytochrome P450 [Postia placenta] |
| Unigene35109_All | 1 | 216 | cytochrome P450 [Postia placenta] |
| Unigene17017_All | 220 | 174 | cytochrome P450 [Dichomitus squalens LYAD-421 SS1] |
| Unigene34280_All | 4 | 133 | cytochrome P450 [Postia placenta] |
| Unigene10849_All | 313 | 195 | cytochrome P450 [Dichomitus squalens LYAD-421 SS1] |
| Unigene30764_All | 16 | 6 | cytochrome P450 [Trametes versicolor FP-101664 SS1] |
| Unigene3813_All | 316 | 466 | cytochrome P450 [Postia placenta] |
| Unigene24466_All | 90 | 36 | cytochrome P450 [Dichomitus squalens LYAD-421 SS1] |
| Unigene20273_All | 28 | 69 | cytochrome P450 [Postia placenta] |
| Unigene35190_All | 103 | 374 | cytochrome P450 [Trametes versicolor FP-101664 SS1] |
| Unigene28522_All | 284 | 636 | cytochrome P450 [Postia placenta] |
| Unigene19978_All | 52 | 153 | cytochrome P450 [Postia placenta] |
| Unigene1072_All | 18 | 10 | cytochrome P450 [Postia placenta] |
| Unigene6094_All | 4 | 10 | cytochrome P450 [Postia placenta] |
| Unigene2796_All | 25 | 41 | cytochrome P450 [Postia placenta] |
| Unigene6403_All | 41 | 1310 | cytochrome P450 [Postia placenta] |
| Unigene2716_All | 27 | 13 | cytochrome P450 [Postia placenta] |
| Unigene19772_All | 686 | 511 | cytochrome P450 [Postia placenta] |
| Unigene9550_All | 54 | 1310 | cytochrome P450 [Postia placenta] |
| Unigene20618_All | 131 | 253 | cytochrome P450 [Postia placenta] |
| Unigene36084_All | 0 | 6 | cytochrome P450 [Postia placenta] |
| Unigene15296_All | 51 | 18 | cytochrome P450 [Postia placenta] |
| Unigene352_All | 72 | 48 | cytochrome P450 [Postia placenta] |
| Unigene7015_All | 143 | 151 | cytochrome P450 [Dichomitus squalens LYAD-421 SS1] |
| Unigene753_All | 8 | 6 | cytochrome P450 [Postia placenta] |
| Unigene5670_All | 41 | 104 | cytochrome P450 [Postia placenta] |
| Unigene36459_All | 4 | 380 | cytochrome P450 [Postia placenta] |
| Unigene5672_All | 52 | 157 | cytochrome P450 [Postia placenta] |
| Unigene754_All | 49 | 36 | cytochrome P450 [Postia placenta] |
| Unigene10816_All | 38 | 183 | cytochrome P450 [Dichomitus squalens LYAD-421 SS1] |
| Unigene15925_All | 34 | 32 | cytochrome P450 [Postia placenta] |
| Unigene10815_All | 4 | 12 | cytochrome P450 [Dichomitus squalens LYAD-421 SS1] |
| Unigene15969_All | 244 | 235 | cytochrome P450 [Postia placenta] |
| Unigene15926_All | 109 | 245 | cytochrome P450 [Postia placenta] |
| Unigene20274_All | 562 | 2239 | cytochrome P450 [Trametes versicolor FP-101664 SS1] |
| Unigene1188_All | 927 | 283 | cytochrome P450 [Postia placenta] |
| Unigene15797_All | 250 | 454 | cytochrome P450 [Postia placenta] |
| Unigene15796_All | 3 | 15 | cytochrome P450 [Postia placenta] |
| Unigene16603_All | 308 | 783 | cytochrome P450 [Postia placenta] |
| Unigene17976_All | 27 | 240 | cytochrome P450 [Trametes versicolor FP-101664 SS1] |
| Unigene15298_All | 196 | 61 | cytochrome P450 [Postia placenta] |
| Unigene22047_All | 10142 | 76659 | cytochrome P450 [Postia placenta] |
| Unigene6067_All | 175 | 180 | cytochrome P450 [Postia placenta] |
| Unigene689_All | 483 | 2765 | cytochrome P450 [Postia placenta] |
| Unigene40192_All | 0 | 5 | cytochrome P450 [Postia placenta] |
| Unigene35055_All | 8 | 474 | cytochrome P450 [Postia placenta] |
| Unigene8021_All | 40 | 119 | cytochrome P450 [Postia placenta] |
| Unigene39952_All | 3 | 8 | cytochrome P450 [Postia placenta] |
| Unigene7550_All | 48 | 27 | cytochrome P450 [Phanerochaete chrysosporium] |
| Unigene31632_All | 20 | 4 | cytochrome P450 [Postia placenta] |
| Unigene34293_All | 0 | 18 | cytochrome P450 [Postia placenta] |
| Unigene17920_All | 19 | 418 | cytochrome P450 [Postia placenta] |
| Unigene35056_All | 0 | 10 | cytochrome P450 [Dichomitus squalens LYAD-421 SS1] |
| Unigene12199_All | 9 | 21 | cytochrome P450 [Postia placenta] |
| Unigene34294_All | 0 | 2 | cytochrome P450 [Postia placenta] |
| Unigene34130_All | 8 | 37 | cytochrome P450 [Postia placenta] |
| Unigene17237_All | 235 | 671 | cytochrome P450 [Postia placenta] |
| Unigene25397_All | 156 | 30 | cytochrome P450 [Postia placenta] |
| Unigene13174_All | 10 | 66 | cytochrome P450 [Punctularia strigosozonata HHB-11173 SS5] |
| Unigene36628_All | 112 | 16 | cytochrome P450 [Trametes versicolor FP-101664 SS1] |
| Unigene36627_All | 12 | 131 | cytochrome P450 [Postia placenta] |
| Unigene19903_All | 121 | 214 | cytochrome P450 [Postia placenta] |
| Unigene6389_All | 1 | 43 | cytochrome P450 [Trametes versicolor FP-101664 SS1] |
| Unigene564_All | 16 | 40 | cytochrome P450 [Postia placenta] |
| Unigene6388_All | 13 | 233 | cytochrome P450 [Trametes versicolor FP-101664 SS1] |
| Unigene10041_All | 29 | 858 | cytochrome P450 [Dichomitus squalens LYAD-421 SS1] |
| Unigene1903_All | 212 | 711 | cytochrome P450 [Postia placenta] |
| Unigene3847_All | 2 | 8 | cytochrome P450 [Postia placenta] |
| Unigene18697_All | 1059 | 1434 | cytochrome P450 [Postia placenta] |
| Unigene18241_All | 36 | 27 | cytochrome P450 [Postia placenta] |
| Unigene1449_All | 2 | 14 | cytochrome P450 [Dichomitus squalens LYAD-421 SS1] |
| Unigene1448_All | 0 | 61 | cytochrome P450 [Dichomitus squalens LYAD-421 SS1] |
| Unigene5704_All | 51 | 653 | cytochrome P450 [Postia placenta] |
| Unigene21395_All | 33 | 3572 | cytochrome P450 [Dichomitus squalens LYAD-421 SS1] |
| Unigene5960_All | 369 | 542 | cytochrome P450 [Postia placenta] |
| Unigene25117_All | 13 | 32 | cytochrome P450 [Postia placenta] |
| Unigene20617_All | 59 | 119 | cytochrome P450 [Postia placenta] |
| Unigene14381_All | 222 | 40 | cytochrome P450 [Postia placenta] |
| Unigene15380_All | 25 | 7 | cytochrome P450 [Postia placenta] |
| Unigene31523_All | 11 | 12 | cytochrome P450 [Phanerochaete chrysosporium] |
| Unigene34538_All | 10 | 19 | cytochrome P450 [Postia placenta] |
| Unigene1784_All | 167 | 151 | cytochrome P450 [Postia placenta] |
| Unigene21055_All | 392 | 1082 | cytochrome P450 [Postia placenta] |
| Unigene11644_All | 620 | 2656 | cytochrome P450 [Phanerochaete chrysosporium] |
| Unigene36082_All | 5 | 157 | cytochrome P450 [Postia placenta] |
| Unigene6199_All | 72 | 10 | cytochrome P450 [Postia placenta] |
| Unigene6198_All | 141 | 114 | cytochrome P450 [Postia placenta] |
| Unigene27582_All | 17 | 11 | cytochrome P450 [Postia placenta] |
| Unigene353_All | 6 | 4 | cytochrome P450 [Postia placenta] |
| Unigene354_All | 32 | 18 | cytochrome P450 [Postia placenta] |
| Unigene25288_All | 50 | 32 | cytochrome P450 [Postia placenta] |
| Unigene19072_All | 20 | 65 | cytochrome P450 [Auricularia delicata TFB-10046 SS5] |
| Unigene17582_All | 18 | 11 | cytochrome P450 [Trametes versicolor FP-101664 SS1] |
| Unigene2724_All | 26 | 16 | cytochrome P450 [Trametes versicolor FP-101664 SS1] |
| Unigene35231_All | 4 | 20 | cytochrome P450 [Postia placenta] |
| Unigene35230_All | 290 | 34 | cytochrome P450 [Postia placenta] |
| Unigene24011_All | 1116 | 115 | cytochrome P450 [Postia placenta] |
| Unigene14207_All | 233 | 9173 | cytochrome P450 [Auricularia delicata TFB-10046 SS5] |
